# Apigenin Suppresses the Warburg Effect and Stem-like Properties in SOSP-9607 Cells by Inactivating the PI3K/Akt/mTOR Signaling Pathway

**DOI:** 10.1155/2022/3983637

**Published:** 2022-03-09

**Authors:** Yihua Shi, Kai Lian, Jiguang Jia

**Affiliations:** Department of Orthopaedics, Xiangyang No. 1 People's Hospital, Hubei University of Medicine, Xiangyang 441000, China

## Abstract

Osteosarcoma (OS) is a prevalent primary malignant bone tumor that commonly occurs in children and adolescents. Apigenin (4′,5,7-trihydroxyflavone) is one of the most researched phenolic compounds that exhibits antitumor effects in several cancers. The aim of the current study was to investigate the effect and underlying mechanisms of apigenin on OS. To address this, OS cells (SOSP-9607) were treated with different concentrations of apigenin. The proliferation, migration, invasion, stem-like properties, and Warburg effect of apigenin-treated OS cells were evaluated. Apigenin was found to suppress the proliferation of SOSP-9607 cells and inhibit epithelial-mesenchymal transition, as indicated by decreased number of migrated and invaded cells, decreased protein expression of vimentin, and increased protein expression of E-cadherin. Additionally, apigenin suppressed tumorsphere formation and reduced the proportion of SOSP-9607 cells with positive expression of the stem cell-related markers Nanog and OCT-4. Apigenin inhibited the Warburg effect in SOSP-9607 cells, as demonstrated by decreased glucose and lactic acid levels, increased citrate and ATP levels, and downregulation of GLUT1, HK1, and LDHA, which are metabolism-related enzymes related to the Warburg effect. Moreover, apigenin inhibited the phosphorylation of PI3K, Akt, and mTOR in SOSP-9607 cells. Collectively, these results indicate that apigenin suppresses the Warburg effect and stem-like properties in SOSP-9607 cells, which may be mediated by PI3K/Akt/mTOR signaling, thus, providing a novel strategy for OS treatment.

## 1. Introduction

Osteosarcoma (OS) is a prevalent primary malignant bone tumor that commonly occurs in children and adolescents [1] and accounts for approximately 5% of all cases of childhood cancer [2]. Despite many efforts, such as surgical resection combined with radiotherapy and/or chemotherapy, the five-year survival rate of patients with OS is only 60% because of the high recurrence rate, metastasis, and poor prognosis [3–5]. In particular, OS patients may develop pulmonary metastases within a few months or weeks [6]. Although researchers have focused on finding efficient methods for OS treatment in the past few decades, progress has been slow [5]. Thus, the development of novel strategies for OS therapy is of great importance.

Warburg effect refers to the energy metabolism of cancer cells that depends on anaerobic glycolysis for completion. Anticancer strategies based on the Warburg effect involve compounds and dietary changes [7]. Valproic acid reduces glucose uptake and decreases lactate and ATP production to inhibit aerobic glycolysis in neuroblastoma cells, ultimately inhibiting the Warburg effect and tumor progression [8]. The Warburg effect is a hallmark of cancer cells. Enhanced glucose consumption and lactate excretion promote the Warburg effect, which is associated with oncogenic growth [9]. The Warburg effect is one of the hallmarks of cancer cells and is characterized by enhanced aerobic glycolysis, and the Warburg effect provides a theory of an inhibitory role in tumorigenesis [10]. Galangin reverses the Warburg effect in hepatocellular carcinoma cells, inhibits hepatocellular carcinoma cell proliferation, and is associated with glycolytic pathways [11]. The search for potential treatments for osteosarcoma through the Warburg effect may be a new direction.

In recent years, natural small-molecule compounds have received extensive attention as effective substances for cancer treatment [12, 13]. Apigenin (4′,5,7-trihydroxyflavone), a natural small-molecule compound, is a hypotoxic phenol widely distributed in fruits, vegetables, plant-based beverages, and herbs [14]. The excellent antiproliferative effect of apigenin on the treatment of diseases, such as diabetes, Alzheimer's disease, and cancer, was observed [15–17]. In particular, apigenin exhibits antitumor activities through the regulation of cancer cell apoptosis, stem cell-like properties, and energy metabolism [18–20]. Chen et al. found that apigenin attenuates tumorigenesis in cisplatin-resistant colon cancer cells by promoting programmed cell death and autophagy via the TOR/PI3K/Akt signaling pathway [21]. Apigenin induces apoptosis of hepatocellular carcinoma cells by inactivating PI3K/Akt/mTOR signaling [22] and suppresses the development of triple-negative breast cancer by attenuating the stem cell-like properties of cancer cells [23]. In addition, apigenin decreases the survival and migration of the CD44+ stem cell population in prostate cancer, which may be mediated via the regulation of PI3K/Akt/NF-*κ*B signaling [24]. However, the effect and underlying mechanism of apigenin on OS have not been thoroughly investigated. In the current study, OS cell lines were cultured with apigenin to explore its effects and underlying mechanisms in OS for its treatment.

## 2. Materials and Methods

### 2.1. Cell Incubation and Treatment

OS cell lines, SOSP-9607, U-2OS, and MG-63, and normal bone marrow stromal cells (BMSCs, not immortalized) were supplied by the Shanghai Institutes for Biological Sciences, Chinese Academy of Sciences. SOSP-9607 and U-2OS cells were cultured in RPMI-1640 medium (Hyclone, UT, USA) supplemented with 10% fetal bovine serum (FBS; Gibco, Gaithersburg, MD, USA). MG-63 cells were cultured in Dulbecco's modified Eagle's medium (DMEM; Hyclone) supplemented with 15% FBS. BMSCs were cultured in human MSC serum-free medium (Chinese Academy of Sciences). All cells were maintained at 37°C in 5% CO_2_. To determine the optimal dose and intervention time of apigenin (Aladdin, Shanghai, China), SOSP-9607, U-2OS, MG-63, and BMSCs were treated with different concentrations of apigenin (0, 0.1, 1, 10, 20, 50, or 100 *μ*M) for 24, 48, and 72 h. The cell viability was measured using a cell counting kit-8 (CCK-8) assay.

To investigate the effects and underlying mechanisms of apigenin, SOSP-9607 cells were selected as representative OS cells. SOSP-9607 cells were incubated for 24 h with apigenin (10, 20, or 40 *μ*M) or doxorubicin (0.1 *μ*M; Aladdin) as a positive control. Clone formation assay, transwell assay, flow cytometry, and western blotting were performed to characterize the cells.

### 2.2. CCK-8 Assay

Cells in the logarithmic growth phase were harvested and seeded into 96-well plates at a density of 5 × 10^3^ cells/well with 180 *μ*L of medium. After an overnight incubation at 37°C in 5% CO_2_, the cells were treated with apigenin (0, 0.1, 1, 10, 20, 50, or 100 *μ*M) for 24, 48, and 72 h. Thereafter, 10 *μ*L of CCK-8 solution (Bioswamp, Wuhan, China) was added to each well, and the plates were subsequently incubated at 37°C for 4 h. The absorbance of each well was measured using an AMR-100 apparatus (Allsheng, Hangzhou, China).

### 2.3. Clone Formation Assay

The clone formation assay was performed to determine the effect of apigenin on the proliferation of SOSP-9607 cells. The cells were first treated with apigenin, subsequently seeded into 6-well plates at a density of 5000 cells/well (2 mL/well), and cultured at 37°C in 5% CO_2_. When clones became visible, they were fixed with 4% paraformaldehyde for 15 min at 4°C and subjected to Giemsa staining (Solarbio, Beijing, China) for 20 min. Images were captured using an inverted fluorescence microscope (Leica, Wetzlar, Germany).

### 2.4. Transwell Assay

The transwell assay was performed to evaluate the effect of apigenin on the migration and invasion of SOSP-9607 cells. The harvested cells were starved in serum-free RPMI-1640 medium for 24 h and resuspended in RPMI-1640 medium supplemented with 1% FBS at a density of 1 × 10^5^ cells/mL. The lower transwell chamber was filled with 750 *μ*L of RPMI-1640 medium supplemented with 1% FBS, and 500 *μ*L of cells were seeded into the upper chamber. For the cell invasion assay, the inserts were precoated with Matrigel (BD Biosciences, Shanghai, China). After an 8 h incubation at 37°C, the cells were incubated in RPMI-1640 medium supplemented with 1% FBS and various concentrations of apigenin for 24 h, fixed with 4% paraformaldehyde for 20 min at room temperature, and stained with 0.1% crystal violet (Solarbio) for 30 min. The migrated and invaded cells were counted using an inverted fluorescence microscope (Leica).

### 2.5. Tumorsphere Formation

After 24 h of apigenin treatment, the cells were collected and resuspended at a density of 2 × 10^3^ cells/mL in DMEM/F-12 (Hyclone) supplemented with 20 *μ*g/mL basic fibroblast growth factor (PeproTech, NJ, USA), 20 ng/mL epidermal growth factor (PeproTech), 5 *μ*g/mL insulin (Solarbio), 2% B27 (Gibco), and 10 *μ*g/mL transferrin (Solarbio). Then, 2 mL of cells were seeded into ultralow adherent 6-well dishes (Corning, NY, USA) and cultured at 37°C in 5% CO_2_ for seven days. Tumorspheres were observed using an inverted fluorescence microscope (Leica).

### 2.6. Flow Cytometry

After apigenin treatment, 1 × 10^6^ cells in each group were harvested and centrifuged at 1500 rpm for 5 min at 4°C, resuspended in 1 mL of fixing agent (BD Bioscience, Franklin Lakes, NJ, USA), incubated in the dark for 20 min, and centrifuged at 1500 rpm for 5 min at 4°C. The cells were resuspended in 1 mL of intraprep permeabilization reagent (BD Bioscience), incubated in the dark for 20 min, and centrifuged at 1500 rpm for 5 min at 4°C. Then, the cells were resuspended in 100 *μ*L of phosphate-buffered saline and incubated with 2 *μ*L of 4-fluorescein isothiocyanate-conjugated antibodies against octamer-binding protein (OCT)-4 (BD, Shanghai, China) and Nanog (Thermo Fisher, MA, USA) in the dark at 4°C for 45 min. Finally, the cells were resuspended in 400 *μ*L of phosphate-buffered saline and subjected to flow cytometry (ACEA Biosciences, San Diego, CA, USA) in the dark at 4°C.

### 2.7. Detection of Glucose Uptake, Lactate, Citrate, and ATP Levels in SOSP-9607 Cells

Glucose uptake (361510) in SOSP-9607 cells was measured using corresponding commercial kits purchased from Shanghai Rongsheng Biotechnology Co., Ltd. (Shanghai, China) following the manufacturer's instructions. Lactate. (A019-2-1) and ATP (A095) levels in SOSP-9607 cells were measured using corresponding commercial kits purchased from Nanjing Jiancheng Bioengineering Institute (Nanjing, China) following the manufacturer's instructions. Citrate levels in SOSP-9607 cells were measured using corresponding commercial kits purchased from Abcam (Cambridge, UK) following the manufacturer's instructions.

### 2.8. Western Blot Analysis

Total proteins were extracted from SOSP-9607 cells using radioimmunoprecipitation assay lysis buffer (Bioswamp) and quantified using a bicinchoninic acid assay kit (Bioswamp). Each sample containing 20 *μ*g of protein was separated and transferred onto polyvinylidene fluoride membranes (Millipore, MA, USA). The membranes were blocked using 5% skim milk and incubated with primary antibodies against glucose transporter 1 (GLUT1, MAB37348, 1 : 1000), hexokinase 1 (HK1, MAB37234, 1 : 1000), lactate dehydrogenase A (LDHA, PAB30703, 1 : 1000), vimentin (PAB40646, 1 : 1000), E-cadherin (PAB43792, 1 : 1000), PI3K (PAB30084, 1 : 1000 dilution), p-PI3K (PAB43641-P, 1 : 1000), Akt (PAB30596, 1 : 1000 dilution), p-Akt (PAB43298-P, 1 : 1000), mTOR (PAB30674, 1 : 1000 dilution), p-mTOR (PAB36313-P, 1 : 1000), or GAPDH (PAB36269, 1 : 1000) for 1 h at room temperature, followed by incubation with goat anti-rabbit IgG (SAB43714, 1 : 20 000) secondary antibodies for 1 h at room temperature. All antibodies were supplied by Bioswamp. GAPDH served as an internal reference.

### 2.9. Statistical Analysis

Data are presented as the mean ± standard deviation. Differences between groups were analyzed using one-way analysis of variance followed by Tukey's test. *P* values < 0.05 were considered to be statistically significant.

## 3. Results

### 3.1. Determination of the Optimal Concentration and Treatment Time of Apigenin

**Figure 1:**
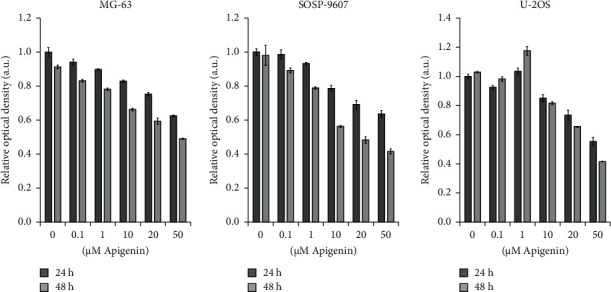
The effect of apigenin on the viability of SOSP-9607, U-2OS, and MG-63 cells was evaluated by the CCK-8 assay. Data represent the mean ± standard deviation (*n* = 3).

As shown in Figure S1, apigenin was nontoxic to BMSCs at low concentrations (<1 *μ*M). However, treatment with a high concentration of apigenin (>10 *μ*M) for more than 48 h decreased the viability of BMSCs. Conversely, after 24 h of treatment, apigenin showed toxicity only at 50 *μ*M or greater concentrations. The effect of apigenin on OS cells was examined using the CCK-8 assay (Figure 1). At concentrations up to 10 *μ*M, apigenin decreased the viability of SOSP-9607, U-2OS, and MG-63 cells in a time- and concentration-dependent manner. Hereby, 10, 20, and 40 *μ*M of apigenin and 24 h of treatment were selected as the conditions for the subsequent experiments. Additionally, the SOSP-9607 cell line was chosen as a representative OS cell line.

### 3.2. Apigenin Attenuated the Proliferation and Epithelial-Mesenchymal Transition (EMT) of SOSP-9607 Cells

**Figure 2:**
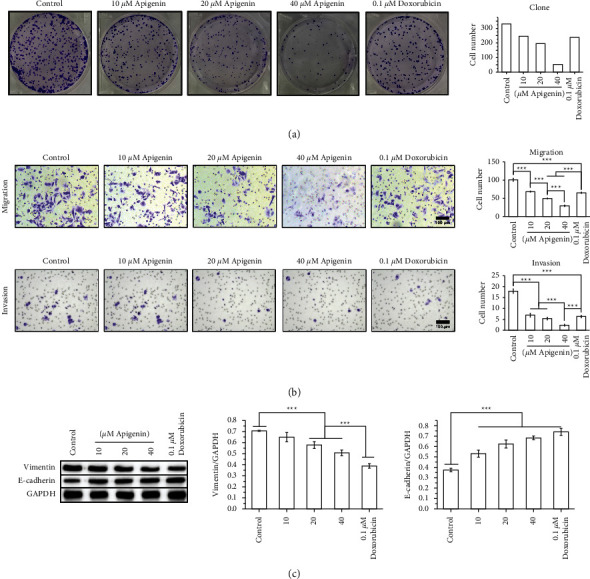
Apigenin attenuates the proliferation and epithelial-mesenchymal transition (EMT) of SOSP-9607 cells. (a) SOSP-9607 cell proliferation was assessed using the clone formation assay. (b) Migration and invasion of SOSP-9607 cells were examined using the transwell assay. (c) Expression of EMT-related proteins (vimentin and E-cadherin) was measured by western blot. Data represent the mean ± standard deviation. (*n* = 3). *∗∗∗P* < 0.001.

The effect of apigenin on the proliferation of SOSP-9607 cells was evaluated by the clone formation assay. As shown in Figure 2(a), apigenin decreased proliferation in a concentration-dependent manner, as demonstrated by decreased colony formation. Furthermore, the effect of apigenin on EMT of SOSP-9607 cells was investigated. The transwell assay revealed that apigenin reduced the number of migrated and invaded SOSP-9607 cells (Figure 2(b)). In addition, apigenin downregulated the protein expression of vimentin and upregulated the protein expression of E-cadherin in a concentration-dependent manner (Figure 2(c)), as indicated by western blotting. The effect of apigenin was similar to that of doxorubicin. These results indicate that apigenin suppressed the proliferation and EMT of SOSP-9607 cells.

### 3.3. Apigenin Attenuated the Stem-Like Properties of SOSP-9607 Cells

**Figure 3:**
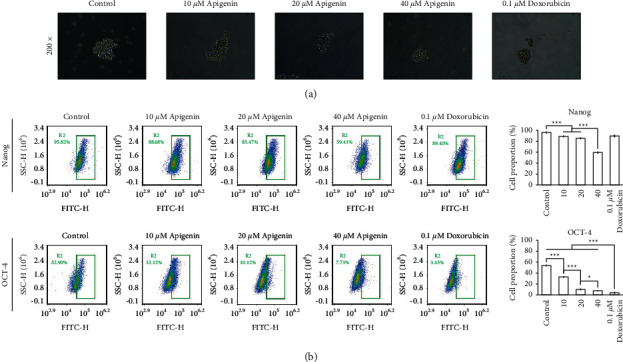
Apigenin attenuates the stem-like properties of SOSP-9607 cells. (a) Tumorsphere formation of SOSP-9607 cells. (b) The proportion of SOSP-9607 cells with positive expression of Nanog and OCT-4 was detected by flow cytometry. Data represent the mean ± standard deviation (*n* = 3). *∗P* < 0.05, *∗∗∗P* < 0.001.

Tumorspheres are solid and spherical structures that are developed from cancer stem cells. As shown in Figure 3(a), apigenin suppressed tumorsphere formation in SOSP-9607 cells. Flow cytometry indicated that apigenin reduced the proportion of SOSP-9607 cells with a positive expression of the stem cell-related markers Nanog and OCT-4 [25] in a concentration-dependent manner (Figure 3(b)). The effect of apigenin was similar to that of doxorubicin. These results suggest that apigenin mitigated the stem-like properties of SOSP-9607 cells.

### 3.4. Apigenin Reduced the Warburg Effect and Activation of PI3K/Akt/mTOR Signaling in SOSP-9607 Cells

**Figure 4:**
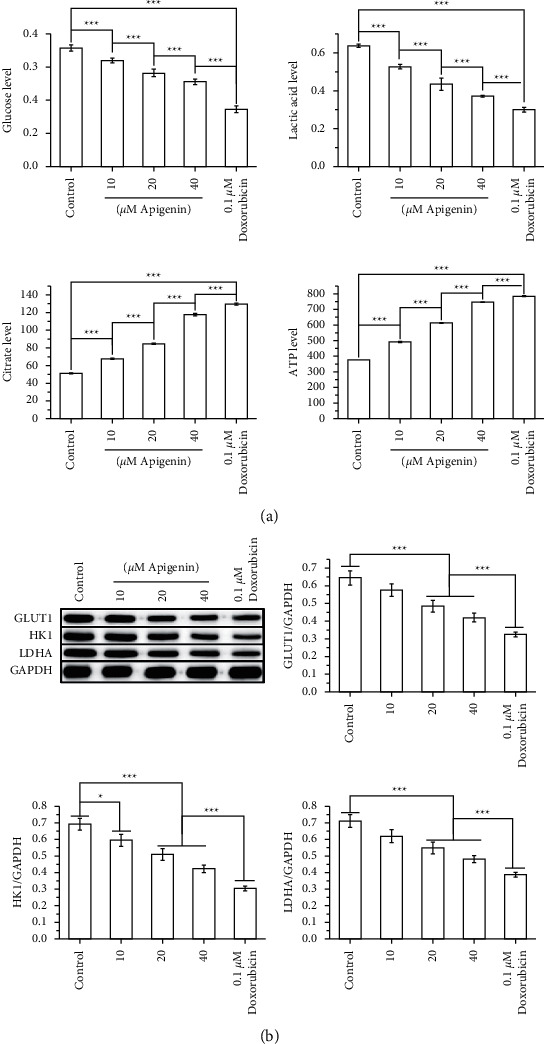
Apigenin suppresses the Warburg effect in SOSP-9607 cells. (a) Levels of glucose, lactic acid, citrate, and APT were measured using corresponding kits. (b) Expression of metabolism-related enzymes associated with the Warburg effect (GLUT1, HK1, and LDHA) was detected by western blot. Data represent the mean ± standard deviation. (*n* = 3). *∗P* < 0.05, *∗∗∗P* < 0.001.

**Figure 5:**
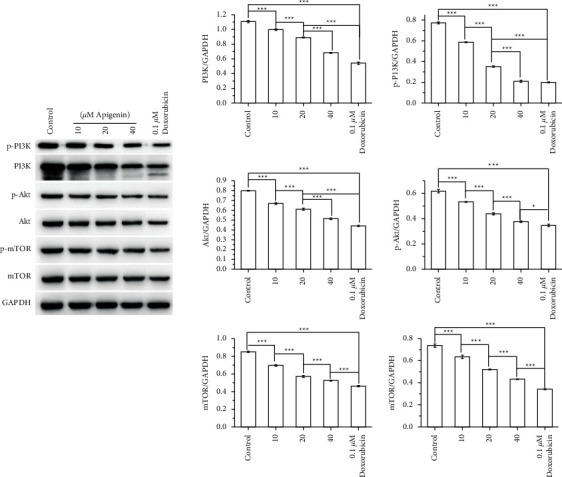
Apigenin inhibits the activation of PI3K/Akt/mTOR signaling in SOSP-9607 cells. The expression and phosphorylation of PI3K, Akt, and mTOR were evaluated by western blot. Data represent the mean ± standard deviation (*n* = 3). *∗P* < 0.05, *∗∗∗P* < 0.001.

As shown in Figure 4(a), apigenin decreased the levels of glucose and lactic acid and increased the levels of citrate and ATP in SOSP-9607 cells in a concentration-dependent manner. Additionally, apigenin downregulated the expression of GLUT1, HK1, and LDHA, which are metabolism-related enzymes associated with the Warburg effect (Figure 4(b)). Moreover, the effect of apigenin on the PI3K/Akt/mTOR pathway was determined by western blotting, which revealed that apigenin reduced the expression of PI3K, Akt, and mTOR and the phosphorylation of PI3K, Akt, and mTOR in a concentration-dependent manner (Figure 5). These results demonstrate that apigenin reduced the Warburg effect and activation of PI3K/Akt/mTOR signaling in SOSP-9607 cells.

## 4. Discussion

Cancer cells prioritize the utilization of glucose for energy via glycolysis, thereby generating plenty of lactic acid through a phenomenon known as the “Warburg effect,” which is a vital form of metabolic reprogramming associated with cancer occurrence and development [26, 27]. Glucose transport from the extracellular environment into cells is the first step in the Warburg effect [28]. Specific membrane transporters, such as GLUTs, are required during the glucose transport process because the hydrophilicity of glucose blocks its transport via simple diffusion [29]. GLUTs such as GLUT1 promote the transport of glucose across the membrane of cancer cells, which in turn enhance glucose utilization [30]. Glucose is then phosphorylated by HK to produce glucose-6-phosphate; this is a critical step in glucose metabolism [31]. Glucose-6-phosphate is subsequently converted into phosphoenol pyruvate through a variety of metabolic enzymes, which is then converted into pyruvate using pyruvate kinase [32]. Finally, pyruvate is converted to lactate by LDH [33]. Upregulation of GLUT1, HK1, pyruvate kinase-M2 splice isoform, and LDHA enhances the Warburg effect in tumor cells [34]. The current study demonstrated that apigenin decreased the levels of glucose and lactic acid and suppressed the expression of GLUT1, HK1, and LDHA. Furthermore, apigenin increased the levels of ATP and citrate, an intermediate in the tricarboxylic acid cycle [35]. It has been suggested that the Warburg effect prevents mitochondrial production of citrate and ATP [36]. Low concentrations of citrate and ATP resist apoptosis and dedifferentiation, thereby promoting tumor aggressiveness [37, 38]. Contrarily, excessive mitochondrial production of ATP and citrate suppresses cellular proliferation [39–41]. Apigenin has shown to suppress the activation of PI3K/Akt/mTOR, a functional pathway that is closely associated with the Warburg effect, in SOSP-9607 cells [42]. It was previously demonstrated that activation of the PI3K/AKT/mTOR signaling pathway accelerates glycolytic flux [43]. Collectively, this study indicated that apigenin attenuated the Warburg effect in SOSP-9607 cells, which might be mediated by PI3K/Akt/mTOR signaling.

Previous studies have demonstrated that the Warburg effect enhances the activity of cancer stem cells (CSCs), which are a subpopulation of cancer cells with stem cell-like features. These cells also possess strong multidirectional differentiation potential, self-renewal ability [44], and enhanced proliferation, growth, and survival, enabling the maintenance of the stem-like state [45]. The capacity for tumor development and reproduction is reportedly associated with the stem-like characteristics of tumor cells [46]. This study found apigenin to suppress the ability of SOSP-9607 cells to form tumorspheres, which are initiated by CSCs. By combining the effects of apigenin on the colony formation ability and proliferation rate of SOSP-9607 cells, it can be found that the inhibition of tumorsphere formation is a manifestation of the inhibition of viability of SOSP-9607 cells, and the inhibition of viability is due to the inhibition of both the proliferation and colony formation ability of tumorsphere cells. In addition, apigenin reduced the proportion of SOSP-9607 cells with positive expression of Nanog and OCT-4, which are stem cell-specific markers [25]. Accumulating evidence has demonstrated an oncogenic function of Nanog and OCT-4 [47, 48], and the inhibition of Nanog and OCT-4 attenuates the stem-like properties such as self-renewal in gastric cancer cells [49]. Chen et al. reported that metformin suppresses the stem-like properties of MG-63 osteosarcoma cells by decreasing the expression of Nanog and OCT-4, thereby suppressing self-renewal and differentiation [50]. As a vital signaling pathway in the delivery of extracellular signals to the nucleus, PI3K/AkT/mTOR is involved in tumor cell growth, apoptosis, and proliferation [51, 52]. This pathway is implicated in CSC-like properties [53] and its inactivation suppresses the self-renewal and survival of glioblastoma-initiating cells by downregulating Nanog and OCT-4 [54]. The present study demonstrated that apigenin attenuated the stem-like properties of SOSP-9607 cells, which might be mediated by PI3K/Akt/mTOR signaling.

Additionally, this study showed that apigenin suppressed EMT of SOSP-9607 cells, as demonstrated by decreased cell migration and invasion, downregulation of vimentin, and upregulation of E-cadherin. In cancer, EMT is related to tumor initiation, metastasis, invasion, and resistance to therapy [55]. The maintenance of stemness of cancer cells and sufficient energy and material metabolism are prerequisites for EMT occurrence, which in turn affects the metabolic reorganization of tumor cells [56, 57].

The potential limitation of current study is that we did not confirm whether apigenin directly inhibited cell proliferation through the altered energy metabolism. In another words, knock down-specific glycolysis enzyme and treatment with apigenin should have been performed to confirm the underlying mechanism, which will be designed in the follow-up study focusing on confirming our current study in vivo.

## 5. Conclusion

In conclusion, the findings of the current study showed that apigenin reduced EMT, stem-like properties, and the Warburg effect in SOSP-9607 cells. The underlying mechanism may be associated with the regulation of the PI3K/Akt/mTOR signaling pathway. This study suggests a novel strategy for the treatment of OS. However, further studies are warranted to investigate whether there is a connection between EMT, the Warburg effect, and stem-like properties.

## Data Availability

The data used to support the findings of this study are included within the article.

## Supplementary Materials

Supplementary Materials Figure S1: toxicity of apigenin on BMSCs was detected by the CCK-8 assay. Data represent the mean ± standard deviation (*n* = 3). ^*∗*^*P* < 0.05, ^*∗∗∗*^*P* < 0.001.
