# Supplementary figures and images for: Apigenin Suppresses the Warburg Effect and Stem-like Properties in SOSP-9607 Cells by Inactivating the PI3K/Akt/mTOR Signaling Pathway
Source: Evid Based Complement Alternat Med. 2022 Mar 9;2022:3983637. doi: 10.1155/2022/3983637 (PMC8926538; doi:10.1155/2022/3983637)

Figure S1


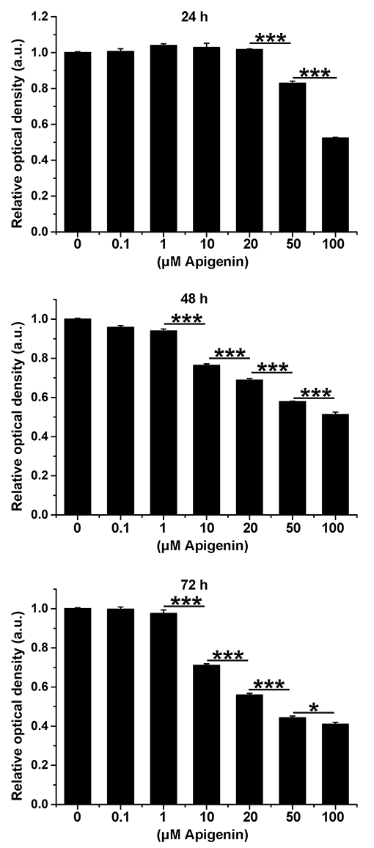

Supplement: Supplementary Materials — Figure S1: toxicity of apigenin on BMSCs was detected by the CCK-8 assay. Data represent the mean ± standard deviation (n = 3). ∗P < 0.05, ∗∗∗P < 0.001. [file 3983637.f1.docx]
